# Modulating the Gut Microbiome in Type 2 Diabetes: Nutritional and Therapeutic Strategies

**DOI:** 10.3390/nu18010089

**Published:** 2025-12-27

**Authors:** Christos G. Nikolaidis, Despoina Gyriki, Elisavet Stavropoulou, Eleni Karlafti, Triantafyllos Didangelos, Christina Tsigalou, Anastasia Thanopoulou

**Affiliations:** 1 Diabetes Center, 1st Propaedeutic Department of Internal Medicine, Medical School, ‘AHEPA’ University General Hospital, Aristotle University of Thessaloniki, 54124 Thessaloniki, Greece; linakarlafti@hotmail.com (E.K.); didang@auth.gr (T.D.); 2 2nd Department of Medicine, Hippokration Hospital, National and Kapodistrian University of Athens, 14564 Athens, Greece; a_thanopoulou@hotmail.com; 3 Hepatogastroenterology Unit, Academic Department of Internal Medicine, General Oncology Hospital of Kifissia “Agioi Anargyroi”, National and Kapodistrian University of Athens, 14564 Athens, Greece; 4 Master Program in “Food, Nutrition and Microbiome”, Laboratory of Hygiene and Environmental Protection, Department of Medicine, Democritus University of Thrace, 68100 Alexandroupolis, Greece; elisabeth.stavropoulou@gmail.com (E.S.); xtsigalou@yahoo.gr (C.T.); 5 Infectious Diseases Service, Department of Medicine, Lausanne University Hospital, University of Lausanne, 1015 Lausanne, Switzerland; 6 Laboratory of Hygiene and Environmental Protection, Department of Medicine, Democritus University of Thrace, Dragana, 68100 Alexandroupolis, Greece

**Keywords:** probiotics, prebiotics, synbiotics, postbiotics, polyphenols, gut microbiota, diabetes mellitus, glycemic control, SCFA, nutritional therapy

## Abstract

Type 2 diabetes mellitus (T2DM) is a complex metabolic disorder influenced not only by genetics, diet, and lifestyle but also by the gut microbiota. Dysbiosis (imbalances in microbial composition) can disrupt gut barrier integrity, alter microbial metabolites, and trigger low-grade inflammation, contributing to insulin resistance and β-cell dysfunction. Nutritional interventions, such as probiotics, prebiotics, synbiotics, postbiotics, and bioactive food components, represent potential therapeutic approaches for ameliorating gut eubiosis and advancing glycemic regulation. This narrative review incorporates evidence from selected studies identified by searches in PubMed, Scopus, and Google Scholar for studies published up to November 2025. The methodology included a structured literature search of in vitro, animal, and human studies, with a focus on intervention trials and mechanistic research. There are many positive signals from randomized controlled trials (RCTs), but heterogeneity and short follow-up limit definitive recommendations. Evidence from clinical and experimental studies indicates a beneficial effect on fasting glucose, hemoglobin A1c, and inflammatory markers, though heterogeneity of the individual and the variability in study designs limit generalization. There is insufficient evidence to recommend microbiota modulation as standard therapy in any disease. Key knowledge gaps include standardized interventions, stratified analyses by medication use (e.g., metformin), clinically meaningful endpoints, and long-term safety data. This review summarizes current knowledge on gut microbiota-driven mechanisms in T2DM and evaluates emerging microbiota-targeted therapies as adjunctive strategies for metabolic improvement.

## 1. Introduction

With approximately 537 million diagnoses across the globe, type 2 diabetes mellitus (T2DM) takes first place among the fastest spreading diseases worldwide [1]. Characterized by insulin resistance, progressive dysfunction of β-cells, and chronic low-grade inflammation, T2DM remains a public health challenge despite the progress in pharmacotherapy and lifestyle modifications. Whereas current treatment focuses on glycemic control, today’s research frontiers are aiming at biological mechanisms that contribute to diabetes onset or progression, including the gut microbiota [2].

The human gastrointestinal tract harbors trillions of microorganisms that constitute the gut microbiome, a dynamic ecosystem essential for the host metabolism, immune regulation, and gut barrier’s integrity [3]. Populations with varying prevalence of T2DM differ in diet, with high fiber and polyphenolic compound-rich diets resulting in high levels of SCFAs [4], while high fat diets characteristic of Western civilization are linked with an imbalanced microbiota and a tendency to develop glucose intolerance [5].

Mounting evidence indicates that dysbiosis, or the imbalance in the gut microbial composition, contributes to increased intestinal permeability, systemic inflammation, and altered glycemic homeostasis, emphasizing the microbiome’s direct effect on glucose metabolism [6,7,8]. Notably, changes in microbial communities can be either a causative factor in the development of T2DM, as well as a consequence of T2DM management with metformin, diet, and glycemic status. This bidirectional connection highlights the complexity of microbiota-host interactions in metabolic disease.

Recognition of the gut microbiome as a modifiable factor of metabolic health has introduced novel opportunities for therapeutic intervention. Numerous approaches to support the balance and diversity of the microbiota, including probiotics, prebiotics, synbiotics, and postbiotics, as well as bioactive substances, are being researched for their influence on glycemia, inflammation, and metabolism. At the same time, perception about the negative impact of antibiotics on gut ecology highlights the urgent need to preserve natural microbiome biodiversity in combating diseases [9,10,11,12,13].

This narrative review explores the role of the gut microbiota dysbiosis in the pathogenesis of T2DM and examines emerging nutritional and therapeutic alternatives aimed at microbiota modulation. It evaluates the therapeutic potential and limitations of probiotics, prebiotics, synbiotics, postbiotics, antibiotics, and bioactive substances from foods like vitamins, polyphenols, polyamines, and mushroom polysaccharides. Discussing consistent findings (e.g., improvements in glycemic index) alongside methological challenges and limitations (e.g., small sample sizes, variable durations), this review aims to provide evidence for understanding microbiome modification as an adjunctive strategy in precision diabetes care and to identify priorities for future research.

## 2. Gut Dysbiosis and Diabetes Pathogenesis

The gut microbiota, comprising trillions of microorganisms in the gastrointestinal tract, is crucial for maintaining human health. It harbors over a thousand microbial species, and the dominant phyla include Firmicutes, Bacteroidetes, Proteobacteria, Actinobacteria, Fusobacteria, and Verrucomicrobia. These microbes play a crucial role in digestion, host metabolism, immune system modulation, resisting enteric pathogen colonization, and overall physiological homeostasis [3]. Microbiome composition and its effects on metabolism vary with host genetics, diet, age, and lifestyle, contributing to inter-individual variability [14]. Dysfunction or gut microbiota imbalance, often referred to as “dysbiosis,” is increasingly recognized as a hallmark of numerous disease conditions [3]. However, owing to the complexity and extreme interindividual variability of microbial communities, there is no gold standard for detection or quantification of such imbalances. This challenge is further compounded by the absence of a definitive characterization of a “healthy” gut microbiota, making it difficult to determine specific microbial deviations linked to disease states and to establish causal relationships [15].

In young, healthy individuals, a healthy gut microbiota produces beneficial metabolites like short-chain fatty acids (SCFAs) (e.g., acetate, butyrate, and propionate) from fermentable carbohydrates; vitamins (such as K, B12, biotin, folic acid, thiamine); peptides with antimicrobial properties (such as defensins and lectins); and secondary bile acids that regulate immune responses and maintain gut integrity [16].

SCFAs affect energy capture and utilization. Acetate is the most common, while propionate aids glucose or lipid biosynthesis. Butyrate protects against insulin resistance and increases energy expenditure in obese mice [17]. Recent studies have shown that the gut microbiota metabolizes primary bile acids into secondary bile acids that activate host receptors [Farnesoid X Receptor (FXR), Takeda G Protein-coupled Receptor 5 (TGR5)] and influence glucose metabolism, e.g., by activating glucagon-like peptide-1 (GLP-1) secretion. Likewise, tryptophan catabolism by bacteria produces indoles (e.g., indole-3-propionic acid), aryl hydrocarbon receptor ligands, and pro-inflammatory regulators. Indole has been shown to enhance GLP-1 release from intestinal L-cells, augmenting insulin release and lowering blood glucose [18].

The gut microbiota plays a pivotal role in diabetes mellitus pathogenesis and progression through multiple interconnected mechanisms (Table 1). Dysbiosis-induced disruption of gut barrier function promotes endotoxin translocation, causing systemic inflammation and insulin resistance. Concurrently, alterations in SCFA production, bile acid signaling, and activation of innate immune pathways, like Toll-like receptors (TLRs) signaling, affect glucose metabolism and contribute to chronic hyperglycemia and metabolic dysregulation. These microbial processes are closely related to both the initiation of insulin resistance and β-cell dysfunction, giving significant insight into the role of the microbiome in metabolic homeostasis [19].

Importantly, the relationship between gut dysbiosis and T2DM is bidirectional. While dysbiosis can contribute to insulin resistance and disease development, hyperglycaemia and diabetes treatments themselves can further modulate gut microbial structure and function [20]. Characteristic microbial signatures associated with T2DM include reduced abundance of certain species such as *Akkermansia muciniphila*, *Faecalibacterium prausnitzii*, and the relative abundance of certain species of *Bacteroides*, which are known to be linked with insulin resistance and intestinal barrier function [21,22].

### 2.1. Increased Gut Permeability

The gut microbiota influences hormone levels and glucose metabolism, leading to hyperglycemia that can enhance bacterial translocation across the intestinal barrier. Bacterial translocation, in turn, causes a chronic inflammatory response by the immune system—a key process that also functions to regulate the composition and function of the gut microbiota to preserve host integrity. However, when translocation of bacteria is exaggerated, it may stimulate inflammation in other organs, causing insulin resistance, fatty liver disease, and beta-cell dysfunction. Ultimately, the complex interaction of all these factors may initiate the development of metabolic disorders [23].

Findings by Mishra et al. show that disrupted ethanolamine metabolism by the gut microbiota in obesity and diabetes leads to ethanolamine buildup, boosting ARID3a-driven miR-101a-3p expression, destabilizing zona occludens-1 (Zo1) mRNA, and weakening intestinal barrier function—boosting permeability, inflammation, and metabolic dysregulation—yet correction of ethanolamine-metabolizing capacity with a new probiotic therapy fixes the ARID3a/miR-101a/Zo1 pathway and restores these adverse outcomes [8].

### 2.2. Small Intestinal Bacterial Overgrowth (SIBO)

SIBO is becoming more prevalent globally, particularly in densely populated cities, and is directly linked to conditions such as diabetes mellitus, irritable bowel syndrome, inflammatory bowel disease, and dysmotility, with recurrent antibiotic exposure further causing bacterial overgrowth through resistance [24].

Diabetic individuals commonly report gastrointestinal disorders and a higher incidence of SIBO than in the general population. A two-way interaction occurs where dysbiosis disables insulin receptors by way of immune stimulation and overwhelming levels of cytokine production. Diabetic complications such as autonomic neuropathy and hyperglycemia weaken motility in the gut, presenting a fertile environment for overgrowth of bacteria [25]. Diabetes mellitus and pancreatic exocrine insufficiency can cause SIBO by reducing the secretion of pancreatic juice, which is typically antimicrobial in nature, and enhancing orocecal transit time, causing neuropathy, opioid use, or maldigestion [26].

### 2.3. Gut-Derived Metabolites

The improvements in glycemia and insulin sensitivity were closely linked with alterations in the gut microbiota-dependent metabolite of trimethylamine N-oxide (TMAO) and its dietary precursors, L-carnitine and choline. The gut microbial metabolites may have modulated the benefits through an alternate pathway involving amino acids linked to diabetes. Dietary fat intake perhaps modified the relations between TMAO, insulin sensitivity, and glucose metabolism improvements [27].

### 2.4. Toll-like Receptor Activation

Toll-Like Receptors (TLRs) are mammalian homologs of the Toll receptor first identified in the course of fruit fly embryonic development and play a critical role in microbial compound recognition, protection from infection, and regulation of sterile inflammation. TLRs, which recognize microbial and host signals, are responsible for immune homeostasis and also function in chronic inflammation [28]. In metabolic diseases such as obesity and T2DM, ongoing low-grade inflammation typically ensues due to TLR activation by Gram-negative bacteria lipopolysaccharides (LPS). Activation leads to the production of reactive oxygen species (ROS) and cytokine signaling, which are aggravated by high-fat diets that reduce numbers of beneficial *Bifidobacterium* and exacerbate inflammation [29].

Toll-like receptor 4 (TLR4), in particular, plays a central role in linking dietary fat intake, gut microbiota changes, and metabolic inflammation. High-fat diets can compromise the integrity of the intestinal barrier, facilitating the translocation of LPS and fatty acids to the circulation, inducing systemic inflammation and insulin resistance [30]. Fatty acids also induce endoplasmic reticulum stress that further stimulates TLR4 signaling [31]. TLR4 is expressed in multiple tissues, including the pancreas, brain, liver, skeletal muscle, and adipose tissue, and impacts insulin sensitivity. However, its overactivation promotes pro-inflammatory cytokine production and oxidative stress, deranging insulin action and leading to metabolic disturbance [32].

Lastly, TLR-mediated interaction of the gut microbiota, diet, and immune system offers potential avenues for the design of targeted therapies in metabolic and autoimmune diseases such as diabetes [33].

## 3. Probiotics

Given the central role of dysbiosis in diabetes, targeting the gut microbiota through probiotics has become an area of intense research.

The term “probiotics” is derived from the Latin word “pro”, meaning “for,” and the Greek word “bios”, meaning “life.” Proposed in 1965, the word “probiotic” was initially used to describe substances produced by one protozoan that stimulated the activity of another. In 1974, Parker expanded the definition to “organisms and substances which contribute to intestinal microbial balance,” emphasizing the impact on the host’s microbiota and including live organisms, as well as non-living materials [34,35]. This definition was subsequently refined to “live microbial feed supplements that improve intestinal microbial balance in the host” and was restricted to exclude non-living substances and antibiotics. The significance of living microbes is emphasized in this new definition, which also removes the ambiguity brought about by using the term “substances” [36]. “Probiotics” now, according to the Food and Agriculture Organization of the United Nations and the World Health Organization, refers to live microorganisms which, when taken in adequate numbers, confer health benefits [37]. The evolution reflects a shift from a general mechanistic understanding to a more precise definition that is focused on the host and the clinical significance. Today, these beneficial microbes are usually administered as lyophilized capsules, and their use is usually advised by medical practitioners—particularly gastroenterologists—who value their ability to support and enhance gastrointestinal health [38].

Functional probiotic strains come from many natural sources like fruits, human-origin foods, and dairy products: breast milk and artisanal cheese. Others are taken from meat products such as Harbin dry sausages, vegetables, fermented foods like kimchi, and dietary supplements. Some were isolated from the gastrointestinal tract of fish and aquaculture settings and some traditional foods, including cucumber jangajji and kefir grains. Italian virgin olive oil bears oil-borne yeast strains, and raw camel milk contains some probiotics, like *Pediococcus acidilactici* [39].

The synthesis of volatile fatty acids, including SCFAs and branched-chain fatty acids (BCFAs), helps maintain energy balance and regulate tissue function within the host. They also exhibit antimicrobial properties by inhibiting the growth of pathogens and inducing the synthesis of bacteriocins. Additionally, probiotics modulate the immune system and gut-associated lymphoid tissue function, as well as intestinal cell adhesion and mucin synthesis. They participate in the metabolism and breakdown of harmful compounds, thereby promoting intestinal and systemic health [40].

Several studies have demonstrated that probiotic supplementation can help modulate and improve symptoms associated with T2DM. Improvements in symptoms result from enhanced intestinal barrier integrity, lowered systemic LPS levels, decreased endoplasmic reticulum stress, and enhanced peripheral insulin sensitivity [41].

In high fructose-induced diabetic rats, the probiotic dahi-supplemented diet containing probiotic *Lactobacillus acidophilus* and *Lactobacillus casei* markedly postponed the onset of glucose intolerance, hyperglycemia, hyperinsulinemia, dyslipidemia, and oxidative stress, suggesting a decreased risk of diabetes and its complications [42].

A meta-analysis of 15 randomized controlled trials (RCTs) and 902 patients found that probiotic treatment significantly improved blood glucose levels in T2DM patients. The results showed reductions in hemoglobin A1c (HbA1c), fasting blood glucose (FBG), and insulin resistance levels [43]. The effect of probiotic intake on glycemic control in individuals with T2DM was also evaluated in the systematic study by Akbari et al. Out of 2736 publications, data from 13 clinical studies where probiotics were used as an intervention and were evaluated by researchers showed that probiotic supplementation effectively lowered HbA1c and fasting blood glucose levels in individuals with diabetes. However, the effectiveness of probiotics was inconsistent with regard to body mass index and the type of probiotics used [44].

Another study showed that giving patients with T2DM 300 g/d of probiotic yogurt containing *L. acidophilus* La5 and *B. lactis* Bb12 enhanced their fasting blood glucose levels and antioxidant status. According to these results, probiotic yogurt may be a functional food with antioxidant and antidiabetic effects [45]. A 2023 meta-analysis of 30 RCTs (1827 patients) showed that probiotics had a clear improvement in glycemic control in T2DM: fasting glucose, HbA1c, insulin, and Homeostatic Model Assessment for Insulin Resistance (HOMA-IR) all reduced compared to placebo [46]. Another review (28 RCTs) showed probiotics reduced fasting glucose by ≈13 mg/dL [47].

The major side effects associated with probiotics involve gastrointestinal effects that are self-limited, skin reactions, stimulation of immune responses, metabolic disturbances, systemic infections (bacteremia, fungemia), sepsis, and endocarditis [48]. The available evidence would therefore suggest cautious assessment of the risk-benefit ratio of probiotics before prescription or recommendation for use [49]. Investigators should monitor probiotic cases and adverse effects, especially in patients with low immunity, premature infants, short bowel syndrome, central venous catheters, and cardiac valve disease [43]. The overall conclusion is balanced: modest benefits have been observed, but heterogeneity and the need for standardized formulations remain key caveats.

## 4. Prebiotics

While probiotics introduce beneficial bacterial strains directly into the gut ecosystem, another complementary approach is to nourish and stimulate the growth of existing beneficial microbes, which is achieved through prebiotics.

Prebiotics, defined in 1995, are non-digestible food ingredients that stimulate the growth and activity of specific bacteria in the colon, improving host health [50]. In 2004, the definition was updated to include three criteria: resistance to gastric acidity, fermentation by intestinal microbiota, and selective stimulation of beneficial intestinal bacteria [51]. By 2010, the definition expanded again to recognize that prebiotics include specific changes in the composition and/or activity of the gut microbiota and that their action can occur at various locations in the host, not only the colon [52]. The International Scientific Association of Probiotics and Prebiotics (ISAPP) is considering the revision of the prebiotic concept. The authors agree on the potential of growth substrates for symbiotic microorganisms in the intestine, but they believe that the concept needs to be revised to strengthen its relevance as a nutritional and therapeutic approach [53].

The identified mechanisms of action of prebiotics in the gut emphasize their roles in immunomodulation, enhanced bowel motility, and improved mineral absorption. Prebiotics, such as inulin, fructooligosaccharides (FOS), and galactooligosaccharides (GOS) promote the growth of beneficial gut microbiota, such as Bifidobacteria, leading to the production of SCFAs, which help regulate immune responses, lower luminal pH, and reinforce the integrity of the intestinal barrier [54,55]. These compounds also influence the secretion of hormones (e.g., GLP-1, peptide YY) involved in appetite regulation while reducing inflammation and decreasing the risk of pathogenic infections [56].

Studies using prospective cohorts have shown that consuming foods rich in prebiotics, such as fruits, vegetables, and whole grains, is clearly associated with a reduced risk of death in both diabetic and non-diabetic groups [57]. In 29 individuals with well-controlled type 2 diabetes, the effects of prebiotic administration were investigated on intestinal microbiota, intestinal permeability, and glucose tolerance. Prebiotic treatment did not substantially alter bacterial populations or clinical outcomes when compared to placebo; however, there was an inverse relationship found between changes in the bacterial family Veillonellaceae, levels of interleukin-6, and glycemic response. Prebiotics may not be useful in treating T2DM since it implies that the heterogeneity of the disease and the use of metformin may be to blame for the absence of substantial changes in the microbiota [58]. The lack of effect in the 29-patient study was in well-controlled diabetics on medication; such findings may not generalize to newly diagnosed or insulin-resistant but non-diabetic individuals.

In a placebo-controlled, randomized study, scientists discovered that a prebiotic (oligofructose-enriched inulin) can specifically change the intestinal flora and dramatically lower body weight z-score, body fat percentage, trunk fat percentage, and serum level of interleukin 6 in children who are overweight or obese [59]. Foods like high-quality extra virgin olive oil contain dietary polyphenols that contribute to the maintenance of gut microbiota, mainly *Lactobacillus* strains, exerting prebiotic actions [60]. A meta-analysis showed prebiotic fiber markedly reduced HbA1c in T2DM (standardized mean difference ≈ −0.43) [46]. Many trials combine prebiotics with other interventions (e.g., diet change), making it hard to isolate effects. Prebiotic efficacy in T2DM may be influenced by baseline microbiome composition and concurrent medications that already alter microbial populations, such as metformin [46]. While prebiotics have a clear mechanistic rationale, consistent clinical efficacy data in T2DM are less robust than for probiotics. Future studies should use standardized fiber preparations and measure both microbiota changes and clinically relevant endpoints (HbA1c, insulin sensitivity).

## 5. Synbiotics

Considering that both probiotics and prebiotics individually contribute to microbiota modulation, combining them offers a synergistic therapeutic potential. Synbiotics are formulations that combine live beneficial microorganisms (probiotics) with selectively fermentable substrates (prebiotics) that support their growth and activity, aiming to promote overall health benefits for the host. There are two subtypes: synergistic synbiotics, whereby the substrate selectively feeds the co-administered microbe, and complementary synbiotics, which pair a probiotic with a prebiotic that targets indigenous microbes, each meeting standard efficacy criteria [61]. Commonly used probiotic strains in synbiotics include species from *Lactobacillus* and *Bifidobacterium*, while typical prebiotic substrates are fructooligosaccharides, galactooligosaccharides, and inulin [62,63]. Synbiotics are being investigated for their potential to modulate the gut microbiota, improve gastrointestinal health, and support immune and metabolic functions [64,65,66].

Synbiotics have been demonstrated to provide an effective modulating impact on the composition of gut microbiota and a positive modification in metabolic factors like insulin sensitivity and glucose homeostasis, regardless of the narrow available studies [17]. Bomhof and colleagues have demonstrated that synbiotic consumption (prebiotic oligofructose and probiotic Bifidobacteria) has beneficial effects on gut microbiota and improved hyperglycemia. A meta-analysis showed improvement in significant clinical markers—such as fasting blood glucose, HbA1c, and lipids- in patients with T2DM who received supplements with probiotics, prebiotics or synbiotics [67]. By improving beneficial bacteria (e.g., *Bifidobacterium*, *Lactobacillus*) and inhibiting harmful strains, they contribute to increased glucose uptake, better lipid metabolism, and pancreatic function [68,69,70].

Another meta-analysis also suggests that synbiotics can improve lipid metabolism and glucose homeostasis in T2DM patients. Nevertheless, due to the limitations inherent in individual trials, they are not yet a direct substitute for conventional therapies, and further research is needed to determine the optimal bacterial strains, doses, and duration [43,53].

The dosage of prebiotics in human studies of their potential benefits usually involves a quantity of several grams of inulin or fructooligosaccharides per day (5–20 g/day), whereas that of probiotics involves a dose of 10^9^ to 10^10^ colony-forming units (CFU) per day [71].

In summary, synbiotics provide synergistic benefits, but they cannot yet replace standard therapy. They are promising but require further RCTs to establish optimal strains, doses, and target populations.

## 6. Antibiotics

In contrast to microbiota-enhancing strategies, antibiotic therapy often disrupts microbial equilibrium and can exacerbate metabolic dysfunction when misused. Antibiotics rapidly alter gut microbiota composition, gene expression, protein activity, and metabolism, leading to increased cell damage, altered active populations, and increased expression of genes related to genetic information processing [72]. The type of antibiotic used and the time of therapy have an impact on the development of diabetes.

Antibiotic exposure is an environmental risk factor for diabetes. Epidemiological data now link cumulative antibiotic exposure (especially in childhood) with rising incidence of both type 1 diabetes mellitus (T1DM) and T2DM, presumably through microbiome disruption [73]. For instance, a Danish case–control study found an antibiotic dose-dependent association with subsequent risk of T2DM. Those with five or more antibiotic prescriptions had 1.53-times higher odds of type 2 diabetes compared to those with 0–1, with slightly higher risks for narrow-spectrum [Odds Ratio (OR) 1.55] and bactericidal (OR 1.48) compared to broad-spectrum (OR 1.31) and bacteriostatic (OR 1.39) types, a clear dose–response pattern, and elevated antibiotic use evident up to 15 years before and after diagnosis [74]. Early-life pulsed therapeutic antibiotic treatment with a β-lactam or macrolide in a mouse model not only accelerated adiposity and insulin resistance in adulthood but also led to chronic alteration of gut community structure and exacerbated high-fat diet–induced metabolic syndrome and glucose intolerance [73,75]. These findings imply that early-life antibiotic disruption may ‘program’ metabolic susceptibility.

Therefore, an antibiotic’s effect on the makeup of the microbiota and a host’s vulnerability to pathogen colonization is partially determined by its range of action and intestine absorption. Among many other things, these can be significant considerations when selecting an antibiotic and forecasting how it will affect the gut microbiome [76,77,78].

Standard antibiotic therapy for infections should not be withheld due to diabetes risk, but unnecessary antibiotic exposure should be minimized. As an example of critical evaluation, it is noted that some commonly used diabetes drugs (e.g., metformin) have microbiome-mediated effects that could interact with antibiotics [74].

## 7. Postbiotics

Another approach to managing glycemic control by modulating the gut microbiota is through postbiotics. In 2019, the ISAPP-convened panel of experts in different fields debated the inconsistent use and undefined term “postbiotics” in scientific papers and commercial products. They agreed that a postbiotic is a preparation of non-viable microorganisms and/or their components (SCFAs, microbial cell wall fragments, bacteriocins, and heat-killed bacterial preparations) that is associated with a health benefit, with the vision of developing a common framework to offer greater regulatory clarity and stimulate future innovation in this area [79,80,81].

Such postbiotics, according to data available, have pleiotropic activity, including immunomodulation, anti-inflammation, anti-diabetic, antioxidant, and anti-cancer effects [82,83,84,85]. They manage metabolic disorders such as obesity and diabetes by enhancing energy expenditure, suppressing fat cell formation and food intake, altering the metabolism of nutrients, and correcting gut dysbiosis [86].

In diabetes mellitus, postbiotics, or bioactive food-grade microorganism-derived compounds, represent novel modulators of gut microbiota and metabolic pathways that can potentially increase insulin sensitivity and minimize inflammation. Although a paucity of existing research exists on specific postbiotic agents such as SCFAs, phenolic acids, and bacteriocins, initial observations and new techniques like fecal transplants portend the advent of new therapeutic strategies in the management of diabetes mellitus [83]. Postbiotics, like SCFAs propionic and butyric acids, play a role in glucose homeostasis through enhancing satiety hormones and inducing intestinal gluconeogenesis, though butyric acid shows limited activity in T2DM patients. Moreover, *Bifidobacterium animalis* exopolysaccharides (EPSs) may improve insulin sensitivity, as shown in animal studies, without affecting fasting glucose to any great extent [87].

Findings revealed camel milk and postbiotic-producing *Lactobacillus brevis* strains (KLDS1.0727 and KLDS1.0373) significantly lowered blood glucose and lipid profiles and preserved liver and kidney function in mice, suggesting their excellent potential as functional food supplements for the management of diabetes and metabolic disorders related to it [88]. One study suggests that postbiotic *Lactiplantibacillus plantarum* LRCC5314 in milk powder should be able to reduce symptoms in stress-T2DM mice by restoring the gut microbiota and SCFAs and altering metabolic and immune gene expression, but this needs to be confirmed for its implications in humans [89]. *Lactobacillus plantarum*-pMG36e-GLP-1 has highly effective therapeutic results in T2DM mouse models through the control of blood glucose, suppression of pancreatic inflammation, and induction of β-cell regeneration. The gene-engineered bacterium also controlled gut microbiota and lipid metabolism in the liver, which represents its potential utility as a new type of T2DM treatment strategy [90].

Postbiotics have also been demonstrated to have beneficial actions on diabetic retinopathy, which may be mediated through various mechanisms: (1) Postbiotics reshape gut microbiota populations. (2) SCFAs not only provide the host with caloric energy but also inhibit histone deacetylases by activating inflammatory signaling. (3) Exopolysaccharides modulate host immunity by activating TLR2/4 in immune cells. (4) Secondary bile acids regulate host metabolism and have neuroprotective effects via G-coupled membrane protein 5 receptor’s activation. (5) Tryptophan catabolites regulate host metabolism and inhibit inflammation [91].

Human studies on postbiotics in type 2 diabetes mellitus are, in fact, limited, and no RCTs exist that firmly support the efficacy of postbiotic supplements in this population [92]. The conclusion is therefore drawn that much of this evidence is preclinical. Until human trials are completed, it is premature to recommend specific postbiotic supplements for T2DM.

**Figure 1:**
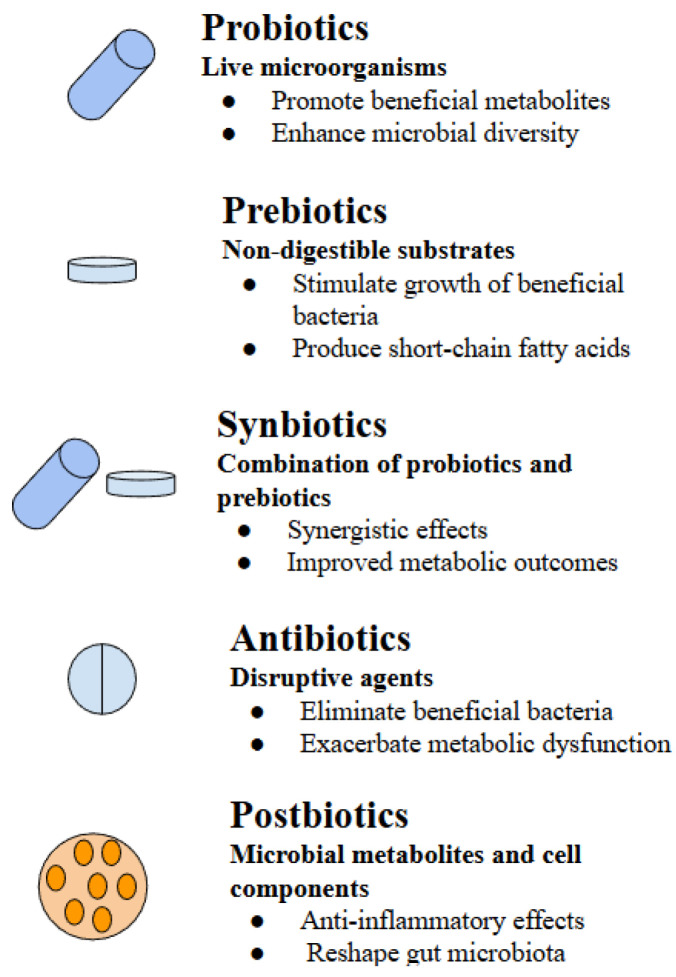
Overview of microbiota-targeted therapeutic strategies for modulating gut health in Type 2 Diabetes. Probiotics, prebiotics, synbiotics, and postbiotics enhance metabolic balance, while misuse of antibiotics causes disruption in microbial diversity.

Overview of microbiota-targeted therapeutic strategies for modulating gut health in Type 2 Diabetes is summarized in Figure 1, while the key metabolic effects induced by probiotics, prebiotics, synbiotics, antibiotics, and postbiotics are summarized in Table 2.

## 8. The Combined Role of Vitamins, Polyamines, Polyphenols and Mushroom Polysaccharides

Beyond direct microbial interventions, dietary components themselves profoundly influence gut ecology and metabolic regulation. Nutritional vitamins, polyamines, polyphenols, and mushroom polysaccharides highlight the strong relationship among nutrition, gut microbiota, and T2DM. Vitamins, particularly B12 and A, regulate microbial activity and glucose metabolism, while deficiency worsens metabolic and vascular outcomes [93]. Polyamines and arginine augment intestinal and metabolic performance, polyphenols have antioxidant, anti-inflammatory, and GLP-1–modulating effects, and mushroom polysaccharides reorganize gut microbiota toward insulin sensitivity and reduced inflammation [94,95,96,97]. In combination, these drugs illustrate the potential for nutrient–microbiota interactions to complement diabetes management and warrant further clinical evaluation.

**Figure 2:**
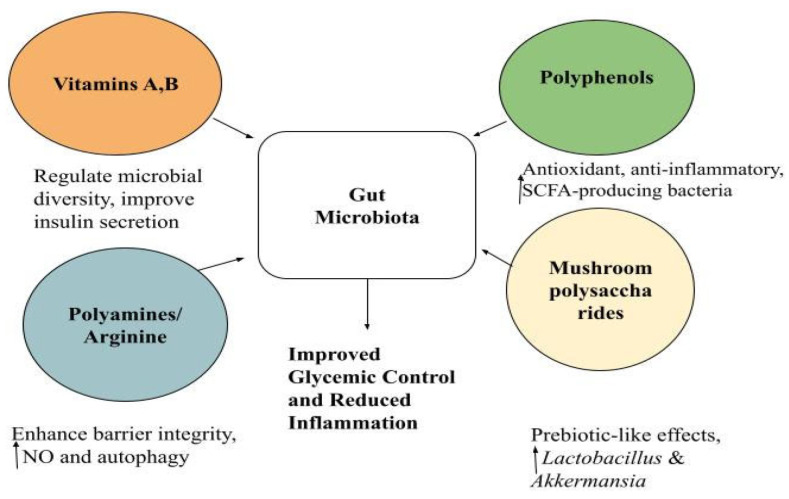
Nutritional modulators of gut microbiota and glycemic control: Vitamins (A, B), polyphenols, polyamines/arginine, and mushroom polysaccharides positively modulate gut microbiota, increasing short-chain fatty acid production, enhancing barrier integrity, and reducing inflammation, hence improving glucose metabolism and insulin sensitivity. NO: nitric oxide, SCFA: short-chain fatty acids, ↑ indicates an increase.

Figure 2 summarizes the collective influence of these compounds on gut microbiota composition and metabolic homeostasis, highlighting their convergent actions toward improved glycemic control and reduced inflammation.

### 8.1. Vitamins

Plant-based diets offer safeguarding against chronic diseases such as cardiovascular disease, cancer, diabetes, obesity, and osteoporosis and support rich, anti-inflammatory gut microbiota. In addition to requiring attention to nutrients like vitamin B12, calcium, vitamin D, iron, zinc, and omega-3s, these diets are capable of being nutritionally adequate at all stages of life using careful food choices and, where necessary, fortified foods or supplements [98].

Vitamin B12 deficiency not only hinders methionine and nucleotide synthesis, leading to homocysteine buildup, immune imbalance, and atherosclerosis promotion, but also exacerbates ischemic stroke risk and severity. In addition, because B12 organizes gut microbial communities and their metabolites, its deficiency can foster dysbiosis-driven immune dysfunction that is involved in stroke pathogenesis and patient recovery [99].

In parallel, the vitamins thiamin (B1) and biotin (B7) (whose synthesis and absorption are modulated by commensal bacteria) function as critical cofactors in carboxylation and carbohydrate-oxidation reactions, so that the microbiota-mediated bioavailability of these vitamins has direct effects on lipid storage, gluconeogenesis, insulin secretion, and vascular function, with possible relevance to glycemic control [93].

Using age- and sex-adjusted linear models in 58 health traits, Wang et al. noted 199 significant correlations of gut microbial vitamin pathways with diabetic traits: B2 and B9 biosynthetic pathways were inversely correlated with HOMA-IR, insulin, glucose, T2DM status, low density lipoprotein (LDL)-cholesterol, triglycerides, C-reactive protein (CRP), and body mass index (BMI), whereas B1 and B12 pathways remained positively correlated with HOMA-IR and insulin levels. Furthermore, consumption of fruits lowered HOMA-IR and insulin through microbial B1 and B2 synthesis modulation and drug exposures—metformin and laxatives—were independently related to increased microbial B1/B6 biosynthesis [100].

Vitamin A, comprising retinol and provitamin-A carotenoids, is needed for cell differentiation, organ formation, and immunity. Deficiency may lead to diminished vision, anemia, and higher infection risk over a lifetime. Primarily through its active metabolite retinoic acid, it seems to preserve epithelial integrity, control glucose homeostasis by adipocyte thermogenesis, and gut barrier–immune interactions in collaboration with specific gut microbiota, and thereby impacts insulin sensitivity and metabolic health [101,102,103]. Although numerous studies have explored the link between dietary vitamin A intake and T2DM risk, their findings remain inconclusive [104,105,106]. Mechanistic and animal studies imply that impaired retinoid signaling diminishes pancreatic β-cell mass and insulin secretion, and dysregulated retinol-binding protein 4 contributes to diabetic development [107].

### 8.2. Polyamines and Arginine Supplementation

Polyamines like putrescine, spermidine, and spermine are crucial to cell function and are regulated by a balance of synthesis, degradation, and transport across the plasma membrane—imbalances of which are associated with cancer and senescence [108]. Recent findings suggest that polyamines from diet or microbiota can promote longevity, improve glucose and insulin sensitivity, and reduce obesity-related pathologies, suggesting that they have potential therapeutic importance through dietary or microbial modulation [109].

Spermidine supplementation has been shown to promote lifespan and healthspan in animal models by inducing autophagy and possibly other mechanisms like enhanced arginine bioavailability and nitric oxide (NO) generation. Due to its low toxicity, endogenous diet-derived occurrence, and promising effect on aging tissues like the heart and kidneys, it is a suitable agent for clinical testing, with ongoing research exploring dietary, supplement-form, and microbiota-directed delivery regimens [110,111]. Spermidine consumption is inversely correlated with obesity and enhances insulin resistance in obese mice by promoting intestinal barrier function and lowering metabolic endotoxemia, possibly via autophagy and TLR4-mediated microbial signaling. It also modifies gut microbiota composition, specifically boosting SCFA-producing *Lachnospiraceae* NK4A136, indicating that spermidine can potentially serve as a novel therapeutic intervention for obesity [112].

Arginine is a key amino acid at the interface of host-microbiota interactions, whose metabolism affects both host immune responses and microbial composition. In the gut, it is used as a substrate by microbes for growth, energy production, and protein synthesis, while its availability can alter the balance between commensal and pathogenic bacteria [113].

In a double-blind, placebo-controlled trial involving 30 young healthy men, oral arginine supplementation (10 g) minimally affected serum growth hormone, thyroid-stimulating hormone, and glucose levels when compared to placebo. Notably, responders had higher benzoate metabolite levels and a compound derived from gut microbiota (X-16124), which suggests that specific microbial signatures have the ability to modulate arginine supplementation hormonal response [114]. In another double-blinded study, oral L-arginine supplementation improved both peripheral and hepatic insulin sensitivity in T2DM patients, likely via normalization of the NO/cyclic-guanosine-3′5′-cyclic monophosphate (cGMP) pathway [115].

Furthermore, increased arginase activity in T2DM lowers arginine availability for NO synthesis, which exacerbates vascular problems and endothelial dysfunction. T2DM patients are more likely to experience microvascular and macrovascular problems if their serum arginine levels are lower and their asymmetric dimethylarginine (ADMA) levels are higher [116,117]. Interventions that inhibit arginase, e.g., L-citrulline, may benefit vascular function and insulin sensitivity through raising NO levels [116].

### 8.3. Polyphenols

Polyphenols, heterogeneous polyphenol components of the plant kingdom with structures including flavonoids, lignans, stilbenes, and phenolic acids, are considered to be essential components of functional foodstuffs because of their health-enforcing properties. When consumed in dietary products like tea, cocoa, vegetables, and fruit, they induce a reduction in oxidative stress and inflammation, resulting in a healthier cardiovascular and metabolic status along with diabetes prevention [118].

Over the past ten years, there has been an increasing number of in vitro and in vivo evidence for the health-promoting activities of polyphenols, including anti-inflammatory, antioxidant, anti-microbial, anti-carcinogenic, anti-adipogenic, anti-diabetic, cardio- and neuro-protective activities [119]. Polyphenols have exhibited antioxidant and anti-inflammatory effects within the brain–liver–gut axis by decreasing inflammation at multiple points in the inflammatory cascade. They also influence key pathways implicated in energy metabolism and adipogenesis, including AMP-activated protein kinase, peroxisome proliferator-activated receptor γ, peroxisome proliferator-activated receptor-gamma coactivator 1-alpha, sirtuin 1, and NF-κB, and thereby the metabolic and immune homeostasis [120].

Polyphenols have been shown to reduce the development of T2DM as well as obesity by the assistance of β-cell function, regulation of glucose and lipid metabolism, and gut microbiota and inflammatory control. In diabetes, some bioactive polyphenols such as curcumin in turmeric possess a role in preventing lipid accumulation and insulin resistance [121]. They also improve T2DM management through the induction of GLP-1 secretion in the gut, where they first engage. Their suggested mechanism coupled with their ability to regulate gut microbiota and secondary metabolites suggests they may play a role in glucose homeostasis and the preservation of metabolic balance [94]. Due to their complex nature, most dietary polyphenols reach the colon intact and undergo metabolism by gut microbiota (*Bifidobacterium* and *Lactobacillus*) to become bioactive, low-molecular-weight metabolites such as protocatechuic and vanillic acids. These microbiota-derived metabolites, including urolithins of ellagic tannins, were demonstrated to exhibit antioxidant and anti-inflammatory properties capable of preventing or curing type 2 diabetes and its complications [122].

A study by Xia et al. identified 29 polyphenols in a vinegar extract, dominated by 4-hydroxybenzoic acid, ferulic acid, and ethyl ferulate, and demonstrated its efficacy in alleviating T2DM symptoms by lowering blood glucose and lipid levels. Vinegar extract has expressed anti-diabetic activity through inhibition of the TLR4/NF-κB inflammatory cascade, restoring the gut microbiota balance—increasing beneficial bacteria including *Bacteroidetes* and *Lactobacillus*—and increasing the generation of SCFAs in diabetic mice [123].

Although research on phenolic substances like resveratrol has promising anti-diabetic action, the optimal dose and length of treatment are indefinite. Both clinical trials and meta-analyses indicated that high (1 g/day) and low (5 mg twice daily) dosages of resveratrol were able to improve blood glucose, insulin sensitivity, and oxidative stress in type 2 diabetics but not in non-diabetics, pointing towards the need for more specific research [124].

While high doses of polyphenols may interfere with glucose-lowering medications, there could be adverse effects associated with certain populations [125]. Their bioactivity often depends on microbiota-mediated metabolism, which varies with individual microbial composition [126,127].

### 8.4. Mushroom-Derived Polysaccharides

Polysaccharides isolated from mushrooms are becoming potential anti-diabetic agents due to their abundant supply, structural versatility, and range of bioactivities. There are existing methods of extraction and evidence supporting their antioxidant, lipid-reducing, anti-inflammatory, and gut-microbiota-modulating action in diabetes prevention and treatment. Further studies on mechanistic insights into insulin signaling, structure–activity relations, and randomized clinical trials are invited [127].

Edible mushrooms, being fiber-, alkaloid-, polysaccharide- and phenolic-rich, act as prebiotic agents that reorganize the gut microbiome to improve insulin sensitivity. Preclinical in vivo and in vitro data show mushroom polysaccharides inhibit glucose uptake, enhance β-cell mass and insulin signaling, while mushroom-derived terpenoids are insulin sensitizers and α-glucosidase inhibitors [128].

In type 2 diabetic rodent models, *Armillariella tabescens* polysaccharide (a compound isolated from a Chinese medicinal mushroom) reduced fasting blood glucose, improved renal biomarkers, and lowered pro-inflammatory mediators with boosted *Lactobacillus* and *Akkermansia* and lowered *Ruminococcus* [129].

The Food mushrooms such as *Ganoderma lucidum*, *Hericium erinaceus*, *Lentinula edodes*, and *Grifola frondosa* act as prebiotic modulators of gut microbiota by enhancing the Bacteroidetes/Firmicutes ratio, promoting anti-inflammatory and SCFA-producing bacteria population, and maintaining intestinal barrier integrity. Such findings indicate their potential to be used as adjunctive therapies to be utilized as a component of microbiota-focused treatment in various clinical conditions [130]. Animal studies have demonstrated that *Ganoderma lucidum* reduced blood glucose and insulin and altered microbiota—lowering *Aerococcus*, *Ruminococcus*, *Corynebacterium*, and *Proteus* and increasing *Blautia, Dehalobacterium, Parabacteroides*, and *Bacteroides* [131]. Finally, *Grifola frondosa* initiated insulin signaling (Insulin Receptor Substrate-1, Phosphoinositide 3-kinase, Glucose Transporter Type 4), downregulated Jun N-terminal Kinase 1/2, and changed the gut microbiota to an increased Bacteroidetes : Firmicutes ratio with decreased Proteobacteria [132].

## 9. Discussion

The role of the gut microbiota in T2DM pathogenesis has shifted our understanding of diabetes from a single-factor metabolic model to a multifaceted host–microbe–environment interaction model [133,134]. The current review emphasizes that dysbiosis, increased intestinal permeability, shifts in microbial metabolites, and low-grade inflammation are cofactors that play an important role in insulin resistance and glucose metabolism modulation. The new landscape sets the gut microbiota as a potentially game-changing treatment approach in T2DM [15,135,136]. Yet, the role of the gut microbiome in T2DM is far more complex and remains incompletely understood [133,137]. Importantly, its influence extends beyond the gut microbiota and the predominantly studied bacteriome [138]. Microbiota-targeted interventions complement pharmacological therapies (e.g., metformin, GLP-1 analogs and SGLT2 inhibitors) but differ mechanistically and depend on host diet and microbial context [139,140]. Even the bacteriome itself represents a vast and heterogeneous community that has not been fully characterized [141]. Also, other microbiome components, such as the virome [142] and mycobiome [143,144], also remain insufficiently explored and were not examined in the present review.

Numerous clinical trials have proven that probiotic supplementation can slightly improve glycemic parameters in T2DM patients, with reductions in fasting blood glucose, HbA1c levels, and markers of inflammation [68,69,145]. Current evidence indicates that probiotics should be used cautiously in immunocompromised patients, with thorough evaluation of potential benefits against associated risks [146]. However, heterogeneity in probiotic strains, dosages, and study durations complicates direct comparisons across studies. Inulin and fructo-oligosaccharides are prebiotic fibers that have demonstrated potential in promoting the growth of beneficial bacteria such as *Bifidobacterium* and increasing the production of SCFAs [147], indirectly improving insulin sensitivity and the function of the intestinal barrier [71,148,149,150]. Synbiotic formulations that combine probiotics with complementary prebiotics may offer synergistic effects [150], even though the available proof is not yet sufficient due to the small scale of the research and brief observation time [62,63].

Other than typical probiotics and prebiotics, latest studies on postbiotics (metabolites or cell components of microbes) indicate that they can be a viable option for precise adjustments of host-microbe relationships without the necessity of sustaining live organisms [92,151,152]. Polyphenols present in berries, tea, and cocoa have a similar impact to prebiotics by selectively promoting the growth of friendly bacteria [153,154,155,156], whereas polysaccharides from mushrooms have been associated with decreased levels of inflammatory cytokines and better lipid profiles [157].

## 10. Future Directions and Personalized Nutrition

Despite the accumulating evidence on the role of gut microbiota modulation T2DM, certain limitations still exist. Current research is highly heterogeneous in terms of design, sample size, type of intervention, and study duration, and it is difficult to draw solid conclusions. Most clinical trials are short-term and have small study populations, which restricts the generalizability of the findings. Moreover, interindividual variability in microbiome composition renders it difficult to predict therapeutic response. Most trials did not assess hard clinical outcomes (e.g., development of complications) and rarely stratified participants by baseline microbiota or medication. Standardized protocols, validated biomarkers, and long-duration randomized controlled trials are still required [158]. Subsequent research should also assign very high priority to precision nutrition approaches that integrate metagenomic, metabolomic, and clinical data to personalize interventions to the individual [159,160]. Therefore, stratification of patients in future trials (by factors like baseline microbiota, diet, medication, age or geography) is crucial. Large, multicenter randomized controlled trials with adequate sample sizes for each specific formulation are needed to assess efficacy, evaluate safety, and inform evidence-based guidelines for the clinical use of probiotics, prebiotics, synbiotics, postbiotics, and other microbiota-targeted therapies in diabetes management [67,68,161].

The future lies in identifying microbial signatures and functional alterations associated with T2DM, which could not only as predictive biomarkers but also inform the development of targeted interventions within a personalized medicine framework [162,163,164,165,166,167]. Moreover, emerging tools such as artificial intelligence can facilitate the interpretation of complex, large-scale datasets, revealing patterns that until recently, were challenging to detect [168]. For instance, machine learning models, such as Random Forest, showed enhanced potential in forecasting glycemic indexes and patient stratification capabilities using microbiome patterns, which aided in formulating personalized nutrition and a therapy plan for type 2 diabetes mellitus [169].

## 11. Conclusions

Nutritional modulation of the gut microbiota is a promising adjunctive therapy for the improvement of glycemic control in T2DM. While probiotics, prebiotics, synbiotics, and food bioactive compounds have shown beneficial metabolic effects, heterogeneous study designs and absence of long-term data limit definitive recommendations. Safety, tolerability and potential adverse effects, especially in immunocompromised or high-risk populations, should be evaluated along with therapeutic efficacy. The next frontier in research involves interventions customized based on individual microbiome profiles, use of outcome measures that are standardized, and studies that delve into the mechanics of the host-microbiome relationship. Integrating microbiome-targeted nutrition into clinical practice will depend on collaborative efforts across disciplines, ultimately advancing precision medicine approaches for diabetes management.

## Figures and Tables

**Table 1:** Gut Microbiota–Diabetes Mechanistic Interactions.

Mechanism	Key Features	Role in Diabetes
Increased Gut Permeability (“Leaky Gut”)	Dysbiosis disrupts gut barrier integrity. LPS is translocated into circulation. Ethanolamine metabolism dysregulation impairs tight junction proteins Zonula Occludens-1 (Z0-1).	Triggers systemic low-grade chronic inflammation and insulin resistance, Promotes β-cell dysfunction and fatty liver disease. Chronic immune activation maintains dysbiosis.
Small Intestinal Bacterial Overgrowth (SIBO)	Overgrowth of bacteria in the small intestine. Common in diabetes due to autonomic neuropathy (reduced motility), altered pancreatic function, and frequent use of antibiotics.	Exacerbates gut dysmotility in diabetic individuals. Inhibits insulin receptor signaling via cytokine overproduction, exacerbating insulin resistance.
Gut-derived Metabolites	Altered production of SCFAs (acetate, propionate, butyrate), TMAO, bile acids, and indoles. SCFAs regulate glucose metabolism, TMAO and bile acids affect insulin sensitivity and GLP-1 secretion.	Reduced SCFA production contributes to insulin resistance. Elevated TMAO impairs glucose metabolism. Indoles and bile acid derivatives modulate incretin release and inflammation.
Toll-Like Receptor (TLR) signaling activation	TLRs detect microbial molecules (e.g., LPS) and regulate immune responses. TLR4 activation connects dietary fat, inflammation, and metabolic dysfunction.	Chronic TLR4 activation promotes metabolic inflammation, β-cell dysfunction, and insulin resistance. Links high-fat diets to gut barrier impairment and systemic inflammation.

GLP-1: glucagon-like peptide-1, LPS: lipopolysaccharides, SCFA: short-chain fatty acids, TLR: Toll-like receptor, TMAO: trimethylamine N-oxide.

**Table 2:** Alterations in Gut Microbiota and Metabolic Effects induced by Probiotics, Prebiotics, Synbiotics, Antibiotics, and Postbiotics. FBG: fasting blood glucose, GLP-1: glucagon-like peptide-1, HbA1c: hemoglobin A1c, SCFA: short-chain fatty acids, IL-6: interleukin-6, T2DM: type 2 diabetes mellitus. ↓ indicates a decrease, ↑ indicates an increase.

Category	Substance/Examples	Key Microbiota and Metabolic Effects
Probiotics	*Lactobacillus acidophilus*, *Lactobacillus casei*, *Pediococcus acidilactici*, *Bifidobacterium lactis* Bb12	Postponed glucose intolerance, reduced hyperglycemia and insulin resistance; ↓ HbA1c and FBG; improved antioxidant status; enhanced intestinal integrity; caution for infections
Prebiotics	inulin, fructooligosaccharides (FOS), galactooligosaccharides (GOS), dietary polyphenols	↑ SCFA production (acetate, butyrate, propionate); immunomodulation; improved barrier integrity; reduced IL-6; weight and fat reduction; selective bacterial stimulation
Synbiotics	Oligofructose + Bifidobacteria, fruit-and-vegetable-enriched diets	Improved hyperglycemia; enhanced insulin sensitivity and glucose homeostasis; improved lipid metabolism; modulation of gut composition
Antibiotics	Early-life β-lactam, macrolide, cumulative antibiotic exposure	Rapid microbiota disruption; dose-dependent ↑ T2DM risk; altered community structure; exacerbated obesity and insulin resistance in rodent models
Postbiotics	SCFAs, phenolic acids, bacteriocins, sonicated *Lactobacillus paracasei*, *O. formigenes* lysates, camel milk-derived, engineered *L. plantarum*-GLP-1	Immunomodulation; anti-inflammatory and antioxidant effects; improved insulin sensitivity; decreased glucose and lipids; β-cell regeneration; gut barrier enhancement

## Data Availability

No new data were created or analyzed in this study. Data sharing is not applicable to this article.
